# Update on overview of ocular manifestations of COVID-19

**DOI:** 10.3389/fmed.2022.877023

**Published:** 2022-09-13

**Authors:** Mitra Akbari, Maryam Dourandeesh

**Affiliations:** Eye Research Center, Department of Eye, Amiralmomenin Hospital, School of Medicine, Guilan University of Medical Science, Rasht, Iran

**Keywords:** SARS-CoV-2, COVID-19, eye manifestations, eye, coronavirus

## Abstract

The *coronavirus* disease 2019 (COVID-19) has become the most critical health crisis at present, and research is continued about the exact pathophysiology, presentations, and complications of this pandemic. It influences several organs, and many studies have addressed the organs, the involvement of which during the COVID-19 results in patients' death. One of the important organs that can be involved during COVID-19, which is also a transmission route of the disease, is the eye. According to the evidence, the severe acute respiratory syndrome coronavirus 2 (SARS-CoV-2) can have ocular manifestations and complications. According to the literature, conjunctivitis is the most common presentation, which can develop at any stage of COVID-19 (during and even after the disease), and the major pathophysiology of the eye involvement during the disease is attributed to the direct effect of the virus on the eyes, tissue damage caused by inflammation, underlying diseases, and the adverse effect of the medications prescribed. There are also reports of life-threatening complications, such as rhino-orbital cerebral mucormycosis, which require urgent treatment and are associated with a great mortality rate. Ocular manifestations may also be the presentation of a life-threatening event, such as stroke; therefore, it is necessary to pay great attention to the ocular manifestations during COVID-19. In this review, after about 2 years of the pandemic started, we present a narrative review on ocular manifestations during COVID-19, categorized into three main categories; ophthalmic, orbital, and neuro-ophthalmological manifestations with a detailed description of the presenting symptoms, risk factor, diagnostic, and therapeutic strategies suggested for each.

## Introduction

The *coronavirus* disease 2019 (COVID-19) was announced as a global pandemic on 11th March 2020 by the World Health Organization, primarily named as 2019 novel *coronavirus* (n CoV) and designated as severe acute respiratory syndrome coronavirus 2 (SARS-CoV-2) by the International Virus Classification Board (1). The pandemic has caused great morbidity and mortality in the world, responsible for the death of more than five million people worldwide, by the time of writing this review (end of 2021), and as a newly emerging disease, research is continued on different aspects of the disease. The SARS-CoV-2 virus mainly affects the respiratory system and presents with cough, difficult breathing, pneumonia, and acute respiratory distress syndrome (ARDS) (2). However, the respiratory system is not the only organ/system affected by the disease; COVID-19 has a wide spectrum of symptoms and complications and can influence almost every organ (3). Therefore, it is necessary for the specialists to have adequate knowledge about the disease manifestations at each specific organ.

The eye is an important organ in COVID-19, as it is one of the routes of disease transmission through hand-eye or aerosol contact with conjunctive, which indicates the necessity of eye protection during the pandemic. More importantly, this causes the ocular manifestations as the early or even the only presentation of COVID-19, although ocular manifestations can be observed at any stage of the disease and even after recovery (4, 5). Since the beginning of the pandemic, several studies, including case reports/series, original research articles, and reviews with or without meta-analysis, have addressed the ocular manifestations of the COVID-19, each with a different perspective. But, COVID-19 has emerged recently, and more studies in this regard can help better identification of different aspects of this disease in order to have a wider perspective about its exact pathophysiology, presentations, and complications and take a step toward more efficient prevention and treatment to reduce its global consequences. Furthermore, the SARS-CoV-2 virus evolves over time and has had several mutations so far, resulting in different subtypes (alpha, beta, gamma, and delta coronaviruses), which can have diverse presentations. Therefore, there is a need for new reviews to present the updated evidence. Accordingly, in this review, after about 2 years of the pandemic started, we present the results of studies that address the ocular manifestations during COVID-19 and categorize the evidence available about each part of the eye for a more clear presentation of the results.

## Methods

A comprehensive search in the online databases, including PubMed, MEDLINE, Science Direct, Scopus, Scielo, and Google Scholar, was performed using different keywords, including “COVID-19” or “SARS-CoV-2” combined with “eye” or “ocular” or “ophthalmology.” Considering the rapidly growing body of the literature, only peer-reviewed reviews and original research articles were included in this study, and case reports, letters, poster presentations, and editorials were not. Human studies were of priority, but when needed, animal studies were also included. Articles with English full text published since 1^st^ January 2020 was evaluated for their appropriateness. Because of the high number of articles on this issue, we attempted to include the most important and unique articles in this review.

## Results

Early studies have described ocular manifestations of COVID-19 as a rare phenomenon (<1%) (6), while more recent studies showed a higher prevalence and the recent meta-analysis of 7,300 patients with COVID-19 reported a pooled prevalence of 11.03% (95% CI: 5.71–17.72) for the ocular manifestations with the most common symptoms, including dry eye or foreign body sensation (16%), eye redness (13.3%), tearing (12.8%), and itching (12.6%) (7). Other ocular diseases and symptoms (such as keratoconjunctivitis/keratitis, scleritis, and neuro ophthalmic manifestations) have also been described by others. In the meta-analysis of 20 case series and case reports (2,228 patients with COVID-19), ocular manifestation was positive in 4.3%, in 0.9% was the first manifestation (8). In a cross-sectional study on 535 subjects, the most common ocular symptoms included conjunctival secretion, blurry vision, foreign body sensation, ocular pain, photophobia, dry eye, itching, and tearing (9). Considering the heterogeneity of studies in terms of the ocular symptoms, a meta-analysis is required for reporting the accurate frequency of each. In the following, we address the different eye segments that can be affected by COVID-19.

### Eyelid, ocular surface, and anterior segment manifestations of COVID-19

#### Ocular surface and cornea

Conjunctivitis, unilateral or bilateral, is the most common ocular manifestation of COVID-19. Prospective studies indicate the overall prevalence of conjunctivitis at about 6% of patients with COVID-19, while retrospective studies suggest a lower rate (<1%) (10). In a meta-analysis of three studies (1167 patients), the frequency of conjunctivitis was higher in patients with severe COVID-19 at admission (3 vs. 0.7% with an odds ratio of 3.4) (11). In a cross-sectional study on 535 subjects, 5% (*N* = 27) presented with conjunctival congestion, four as the initiating symptom (9). In a recent meta-analysis on patients with COVID-19, conjunctivitis was observed in 88.8% of patients with ocular manifestation (7). The direct exposure of the conjunctiva to extraocular pathogens and the connectivity of the ocular surface mucosa and upper respiratory tract (through the nasolacrimal duct) are considered the main mechanisms for direct infection of conjunctival epithelium and the high prevalence of conjunctivitis (12). Furthermore, some have suggested that the expression of angiotensin-converting-enzyme-2 (ACE2) in the conjunctival epithelium, aqueous humor, and retina acts as the receptor-binding motif of SARS-CoV and allows the virus for tissue spread (13). Several symptoms have been reported for COVID-19-related conjunctivitis, including mild symptoms, such as foreign body sensation, conjunctival hyperemia, watery secretions, and itching, to more severe symptoms, such as photophobia, swollen eyelid, mucus secretions, chemosis, dry eye, petechiae, tarsal hemorrhage, and pseudomembrane; some associated with pre-auricular, submaxillary, or cervical lymphadenopathy (14). The timing of conjunctivitis presentation is also variable; some have reported early presentation (presenting as the first symptoms), while late onset conjunctivitis (10–13 days) has also been reported (14).

Ocular transmission of COVID-19 *via* tear is an important issue for disease spread, especially for the protection of ophthalmologists. However, the results of studies are controversial considering the polymerase chain reaction (PCR) results taken from conjunctival swabs or tear samples. In the meta-analysis of 20 case series and case reports, among 412 ocular swabs taken, only 2.9% were positive (*N* = 12), without ocular signs/symptoms in 25% (*N* = 3) (8). Also, in another study on 68 conjunctivitis PCR, 4% (*N* = 3) revealed positive results (15). However, some others have reported negative results, as a Chinese study on 114 Chinese patients, among whom none had ocular symptoms or positive swabs (16). Another study reported the detection rate of the virus in 0–11% of ocular swab samples (17). Nonetheless, the conflicting results considering the presence of virus in the tear does not change the necessity of eye protection for reducing the disease transmission (12, 18, 19). The effective drainage of the ocular surface (as the self-cleaning system) may be responsible for the detachment of the virus from the tear film and pass through the nasolacrimal duct into the nasopharyngeal space. Other factors, such as the outer lipid layer of the tear film and intact ocular surface, presence of antimicrobial agents in tears, and technical difficulties, have been considered responsible for negative PCR results (20).

Conjunctivitis related to COVID-19 in the children is typically mild, presenting with conjunctival discharge, congestion, and eye rubbing, as the initial manifestations of SARS-CoV-2 infection with generally quick recovery and no long-term complications. It does not require treatment in most cases, and inflammatory biomarker abnormalities and lymphocytopenia are also less common findings in children (20). Therefore, COVID-19-related conjunctivitis is generally a benign condition in children.

In addition to conjunctivitis, patients may develop other ocular surface disorders, such as keratitis, pseudomembranous keratoconjunctivitis, conjunctival follicular reaction, episcleritis, hemorrhagic and pseudo-hemorrhagic conjunctivitis; however, these diseases have been only observed in a few case reports (21, 22). Therefore, ophthalmologists are suggested to perform a thorough slit-lamp examination for detection of any ocular surface diseases in patients with COVID-19 (19). Physical examination revealing enlarged preauricular and submaxillary lymph nodes, and the lab data, which show increased serum levels of white blood cell, neutrophil, procalcitonin, C-reactive protein (CRP), lactate dehydrogenase (LDH), and other inflammatory markers, which may accompany the ocular symptoms, can help diagnosis (23).

#### Eyelid

Eyelid manifestation can present as another anterior segment involvement, associated with or without conjunctivitis, presenting with blepharitis, eyelid edema, eyelid dermatitis, meibomian orifice abnormalities, and lid margin hyperemia/telangiectasia. However, the difference in the disease stage and severity, as well as the method of examination and data collection, among studies make a conclusion about the frequency of symptoms difficult (24). In a report on 29 hospitalized COVID-19 patients, 11 cases experienced blepharitis with the symptoms of crusted eyelashes, lid margin telangiectasia and/or hyperemia and altered meibomian orifices (25).

### Posterior segment manifestations of COVID-19

Posterior segment involvement reflects the effect of the virus on vascular, inflammatory, and neuronal properties of the eye, presenting from 4 to 55 days of COVID-19 disease (with a mean duration of 12 days) (24). Optical coherence tomography (OCT) and angiography have been suggested for an accurate eye examination during and after COVID-19 infection, which can detect cotton wool spots (CWS), retinal hemorrhage, central serous retinopathy, papillophlebitis, optic neuritis, optic atrophy, panuveitis, multifocal retinitis, necrotizing retinitis, central retinal artery/vein occlusion, and Purtschner-like retinopathy (26).

#### Retina

The retina can be involved during or after COVID-19 (27), the mechanism of which is supposed to be related to the direct viral ocular infection because of the expression of ACE2 receptors in the retina, as well as the indirect effect of COVID-19 on vascular inflammation and thromboembolic complications (28). Some patients reported positive PCR retinal tests, while others did not (29). As retinal diseases can develop, even with normal visual acuity and in patients with mild to moderate COVID-19, a thorough fundoscopic examination is required for appropriate diagnosis of retinal diseases, during which special attention should be paid to hyper-reflective lesions, cotton-wool spots (CWS), micro-hemorrhage, vascular changes (dilated veins, tortuous vessels) (30). A large series of 54 patients showed that 9.25% of not critically sick patients developed retinal hemorrhage, with CWS found in 7.4% of patients. This study disclosed that retinal involvement is mainly observed in patients with mild to moderate COVID-19 and is associated with elevated serum levels of fibrinogen, CRP, ferritin, and LDH (31). In another study on 18 patients (95% of whom were admitted to the intensive care unit [ICU]), more than half (55.5%) had retinal findings, 22% had a flame hemorrhage, and 5.6% had a macular hemorrhage and hard exudate. This study showed that the diameter of retinal veins is shown to be related to disease severity and negatively to the time from onset of symptoms (32). Others reported CWS and retinal micro-hemorrhages in 4/11 patients (33) and 22% of patients within a median of 43 days from the first symptoms (34). The pathophysiology of retinal changes induced by SARS-CoV-2 has been suggested to be related to the endothelial cell involvement, altered immune and coagulation systems during COVID-19, and the expression of ACE2 on the retina (35). However, in a cross-sectional study on 46 patients affected by severe COVID-19, no case of retinal involvement was observed (36). Therefore, meta-analysis studies are required to report the exact frequency of retinal involvement and its subtypes in patients with COVID-19.

The retinal vascular occlusions, reported in patients with COVID-19, include:

- *Central retinal vein occlusion (CRVO)*: According to the review studies, microvascular changes are the most common vitreoretinal disorder caused by COVID-19 infection (28, 31). Impaired visual acuity was reported as a common complaint, while some were asymptomatic; various findings have also been reported to be found during the fundoscopic examination, such as retinal hemorrhages, and pan-retinal fern-like whitening (37), macular edema (38), dilated and tortuous vessels (39). Nevertheless, investigations, such as fluorescein angiography (FA) and OCT, failed to show diagnostic differences to differentiate CRVO related to COVID-19 from unrelated cases. Special attention should be paid to the hypercoagulation pattern in the patients; a 40-year-old patient presented with bilateral CRVO 5 days after COVID onset, associated with deep vein thrombosis (DVT) in the right leg and right heart strain (40). Therefore, it is important to measure the serum levels of D-dimer, prothrombin time, activated partial prothromboplastin time, fibrinogen, and cytokines in patients without systemic conditions, the elevation of which can refer to the coagulation cascade activated by the COVID-19. Management with steroids and anti-vascular endothelial growth factor, in addition to prophylaxis with anticoagulants, are suggested in the early phase in patients with severe COVID-19 (38).
- *Central retinal artery occlusion (CRAO), acute macular neuroretinopathy (AMN), and paracentral acute middle maculopathy (PAMM):* There is only one case report presenting COVID-19 CRAO; however, as CRAO is an ophthalmic emergency, more attention should be paid to this complication. A 60-year-old man admitted to ICU with elevated inflammatory markers (such as interleukins, CRP, ferritin, fibrinogen, and D-dimer) exhibited a sudden onset of a painless visual impairment with optic disk margin blurring and retinal whitening, 12 days after admission (41). There is also a report of a patient who developed incomplete CRAO while he received enoxaparin for DVT (42). Others have also reported successful diagnosis of PAMM and AMN (which involve deeper retinal vessels) using OCT, indicating hyper-reflective lesions at various sites, while the patient present with an acute painless impaired vision, faintly colorful paracentral scotoma, and dyschromatopsia, and the fundus examination is not conclusive (and may reveal retinal hemorrhages with Roth spots and a wedge-shaped reddish-brown lesion directed toward fovea) (43, 44).
- *Vitritis*: One case presenting with vitritis had bilateral redness in the eyes, and examination showed a yellowish macular lesion 12 days after the onset of COVID symptoms. OCT displayed hyper-reflective lesions at several sites (which could also be suggestive of PAMM/AMN lesions), and FA revealed hyper-fluorescence (45). Definite decision based on OCT findings should be performed, considering the clinical presentations and medical history of patients, to rule out other infectious/inflammatory diseases/agents, such as herpes simplex virus, cytomegalovirus, syphilis, Bartonella, toxoplasma, borrelia, Toxocara, and other diseases that can cause uveitis (24, 35).
- *Acute retinal necrosis (ARN):* it is another retinal involvement during or after COVID-19, with few cases reported. Gupta and colleagues reported an atypical bilateral acute retinal necrosis in a 75-year-old immunosuppressed lady (systemic lupus erythematosus and relapsed diffuse large B cell lymphoma), who received chemotherapy 2 months before ocular involvement; vitreous sample PCR was positive for Varicella Zoster Virus (VZV) (46). Others have also reported reactivation of VZV, presenting as ARN, following COVID-19 infection (47) or vaccination (48, 49). Soni and colleagues also reported two cases of ARN, 1 month after recovery from COVID-19, one was a 5-year-old child with extensive peripheral necrotizing retinitis (treated by oral valacyclovir), and the other was a 61-year-old man with bilateral retinal detachment, sieve-like breaks, and optic atrophy (treated by surgery) (50).

Some of the retinal findings (such as peripheral retinal hemorrhages, macular hyperpigmentation, retinal sectoral pallor, peripapillary flame-shaped hemorrhages, hard exudates, and CWS), in the case reports presented, could not be directly attributed to the effect of the virus because of the presence of underlying diseases, ICU admission, and medicines taken by the patients (31, 32, 46, 51).

#### Choroid and uvea

Chorioretinitis is a common finding in systemic or local infectious and inflammatory conditions and is also reported to be caused by SARS-CoV-2. The inflammatory effect of COVID-19 is supposed as the mechanism of its influence on this highly vascularized tissue, choroid, resulting in chorioretinal inflammation (52). There are very few case reports in this regard, reporting reactivation of serpiginous choroiditis 2 weeks after COVID-19 (53), chorioretinal disease (54), and bilateral ampiginous choroiditis 1 week after SAR-CoV-2 infection (55). There are also few reports of panuveitis during COVID-19 (56–59). There are also reports of panuveitis following COVID-19 vaccination (60, 61). A summary of case presentations of patients with choroiditis and panuveitis, during or after COVID-19, is provided in Table 1.

**Table 1:** A summary of the studies reporting chorioretinal inflammation during or after COVID-19.

**References**	**Study type**	**Number of cases**	**Patients' age and sex**	**Presenting ocular symptoms**	**Interval between ocular and COVID-19 onset**	**Final diagnosis**	**Diagnostic methods**	**Treatment**
**Choroiditis**								
Providencia et al. (53)	Case report	1	40 y, female	Unilateral blurred vision (counting fingers at 2 m)	1 month	Reactivation of serpiginous choroiditis	Fundoscopic examination, OCT, FF, and angiography	High-dose corticosteroid
Ortiz-Seller et al. (54)	Case report	1	51 y, female	ocular pain and difficulty in reading	Concomitant	chorioretinal disease	fundoscopic examination and OCT	60 mg/day prednisone
Tom et al. (55)	Case report	1	25 y, female	Metamorphopsia and a paracentral scotoma	1 week	Bilateral ampiginous choroiditis	Fundoscopic examination and OCT	Starting with 60 mg/day oral prednisone; after 3 weeks azathioprine (1.5 mg/kg)
**Panuveitis**								
François et al. (57)	Case report	1	Late 50s, female	Unilateral blurred vision and redness and temporary pain	2 days	Panuveitis	Fundoscopic examination and FA	Oral and topical corticosteroid
Benito-Pascual et al. (56)	Case report	1	60 y, female	Unilateral ocular pain, blurred vision, and redness	2 weeks	Panuveitis and optic neuritis	Slit-lamp examination and oct	Starting with 60 mg/day oral prednisone, hourly steroids drops, and mydriatics three times per day
Sanjay et al. (58)	Case report	1	66 y, male	Bilateral blurred vision	3 days	Bilateral panuveitis with CRAO in the right eye	Fundoscopic examination	Corticosteroid
Hosseini et al. (59)	Case report	1	37 y, male	Bilateral blurred vision	Recovered	Bilateral neuro-retinitis and panuveitis	Fundoscopic examination	Corticosteroid

FA, Fluorescein angiography; OCT, Optical coherence tomography.

### Neuro-ophthalmic manifestations of COVID-19

Neurological manifestations affecting the central and peripheral nervous systems can develop in patients with COVID-19, with a prevalence of more than one-third of patients reporting signs and symptoms of headache, dizziness, anosmia/hyposmia, ageusia/hypogeusia, muscle damage, ischemic and hemorrhagic stroke (62). Development of neurological syndromes and diseases, such as Guillain-Barre syndrome, encephalitis, acute disseminated encephalomyelitis, polyneuritis, meningitis, and encephalopathy, have also been described during COVID-19 infection (63). Furthermore, the direct role of coronavirus on the central nervous system (CNS) and ophthalmic sequelae, confirmed by animal studies (64). However, some suggest that the virus can affect the neurological system of humans only by the indirect effect and through the increase in proinflammatory cytokines (64). There are only few case reports are available in the literature presenting the neuro-ophthalmic involvement by SARS-CoV-2; the documented conditions include:

#### Optic neuritis

There are few cases of bilateral optic neuritis during COVID-19 infection, both of which report positive anti-myelin oligodendrocyte glycoprotein antibodies, which recommend the immune-mediated demyelination in the optic nerve as the pathogenesis; however, the virus could not be detected in the cerebrospinal fluid (CSF) or on magnetic resonance imaging (MRI) results, which rejects the direct effect of the virus (65, 66). A summary of patients' characteristics, presentation, and treatment is presented in Table 2. Some cases of optic neuritis and panuveitis have also been described earlier and explained in Table 1; thus not repeated in this table (57).

**Table 2:** A summary of the studies reporting optic neuritis during or after COVID-19.

**References**	**Study type**	**Number of cases**	**Patients' age and sex**	**Presenting ocular symptoms**	**Interval between ocular and COVID-19 onset**	**Final diagnosis**	**Diagnostic methods**	**Treatment**
Zhou et al. (66)	Case report	1	26 y, male	Bilateral visual loss, pain with eye movement	Simultaneous	Bilateral optic neuritis and myelitis	Fundoscopic examination, MRI, serology	Corticosteroids (intravenous, followed by oral taper)
Sawalha et al. (65)	Case report	1	44 y, male	Bilateral visual loss, pain with eye movement	1 week	Bilateral optic neuritis	Fundoscopic examination, MRI, serology	Methylprednisolone 1 g/day for 5 days
Catharino et al. (67)	Case report	1	64 y, male	Bilateral visual loss, pain with eye movement	Simultaneous	Optic neuritis	Fundoscopic examination, MRI, serology	Methylprednisolone 1 g/day for 5 days
Parvez et al. (68)	Case report	1	10 y, girl	Visual loss in the left eye	Simultaneous	Optic neuritis	Fundoscopic examination, MRI, serology	Methylprednisolone 1 g/day for 5 days + oral prednisolone
Azab et al. (69)	Case report	1	32 y, male	Left-sided visual impairment and headache	2 weeks	Optic neuritis	Fundoscopic examination, MRI, serology	Methylprednisolone 1 g/day for 3 days + oral prednisolone

MRI, magnetic resonance imaging.

As this table shows, optic neuritis can present at any time of COVID-19 infection; the meta-analysis on five studies in this regard showed more women affected and a higher frequency of visual acuity impairment in the left eye (69); however, definite results can only be reported after publication of more studies reporting optic neuritis associated with COVID-19. Considering the risk of involvement of other nerves, it is strongly recommended to examine CSF and brain MRI in SARS-CoV-2-infected patients. Special attention should also be paid to the differential diagnoses of optic neuritis (70).

#### Miller fisher syndrome and cranial nerve palsy

The frequency of facial nerve palsy, diplopia, and ptosis have been reported to increase after the COVID-19 pandemic (71). Several cases of cranial nerve palsy have been reported in COVID-19 patients (most commonly the 6^th^ nerve, followed by the oculomotor nerve), associated with ocular motor deficits, associated with fatigue, paresthesia, hyporeflexia, ophthalmoplegia, recommending MFS variant of Guillain Barre syndrome (72, 73). Such effect has been suggested to be related to the predisposition to the hypercoagulable and proinflammatory state induced by SAR-CoV-2, which can trigger or exacerbate autoimmune diseases (24, 72). As reported, most cases of cranial nerve palsies were self-limit within about 6 weeks and did not require treatment, and patients with MFS were treated with intravenous immunoglobulin.

#### Visual loss, associated with cerebrovascular accident

Stroke has been an important and life-threatening complication of COVID-19, during which involvement of the posterior circulation and occipital lobes can result in acute vision loss and visual snow syndrome (74). The positive underlying diseases in reported cases suggest that pre-existing endothelial dysfunction may predispose patients to stroke (24). Therefore, examination of double vision pupillary response, ptosis, optic disc, ocular reflexes, and movements, along with gait abnormalities or other neurological conditions, are important in patients with COVID-19.

#### Other neuro-ophthalmological presentations

Several cases of oscillopsia, associated with ataxia and myoclonus, have been reported following severe COVID-19, in the context of encephalitis, documented using cerebral lesions on MRI and/or bland cerebrospinal fluid. The patients were successfully treated with intravenous immunoglobulin and methylprednisolone (75–78).

Few cases have also documented Adie's tonic pupil in COVID-10 patients; one, coexisting with multifocal chorioretinitis, explained earlier (54), which along with other cases of Adie's tonic pupil, reported in the literature (79–81), suggest rare patterns of neurological involvement by SARS-CoV-2. Papillophlebitis is another uncommon ocular involvement in COVID-19 patients, presenting with painless, unilateral, slight diminution of vision, characterized by venous congestion and optic disc edema, tortuous retinal vessels, retinal hemorrhages, and confirmed by OCT and FA results. This condition has also been proposed to be caused by inflammatory and coagulation dysregulation induced by SARS-CoV-2 (82).

### Orbital manifestations of COVID-19

Rhino-orbital cerebral mucormycosis (ROC) is the most common orbital involvement in patients with COVID-19, reported in several case series (83, 84), while other orbital conditions have been reported only in few cases. The main mechanism is the spread from colonization of nasal mucosa. Mucormycosis is a life-threatening infection that can occur in patients with COVID-19, because of the compromised immune system and decreased lymphocytes, due to the disease itself, the underlying diseases (diabetes mellitus and renal failure), decompensated pulmonary function, and treatment with corticosteroids (24, 85).

Mucormycosis is followed by underlying disorders that predisposing the patient. Immunologic changes in diabetic patients have made a potential risk factor for such fungal conditions (86). Reasons why diabetes is associated with increased sensitivity to fungal infections can be attributed to hyperglycemia with impaired range of immune performances in monocytes, neutrophils, macrophages and phagocytosis by eliminating microorganisms and responding to antigens within the cell. Nerve damage and reduced blood flow to tissues in such patients may be important for fungal infections (87).

The COVID-19 induced mucormycosis is more common in patients with diabetes mellitus and suffering from critical or severe COVID-19 (88). Fungi can grow and survive more easily following high blood sugar. Weakened immune systems provide less protection versus infection. Frequently administered antibiotics, steroids and oxygen masks during treatment of severe or critical illness can be risk factors for COVID-19 infection (89).

Most cases have reported ROC after recovery from COVID-19, within 30–42 days after diagnosis. The signs and symptoms of ROC, diagnostic (histopathological and microbiological evidence), and the treatment strategies (antifungal and surgical debridement within 4–72 h) are not different from mucormycosis, and a mortality rate of about 40% has been reported because of the treatment failure (85). Therefore, strong clinical suspicion is required for early diagnosis of ROC, especially in patients with COVID-19 (active or recovered) with facial pain, headache, peri-ocular swelling, and visual impairment (24). Also, special attention should be taken for the appropriate use of corticosteroids for the treatment of COVID-19 in order to reduce the incidence of this complication (85).

Orbital cellulitis and sinusitis have been reported in two adolescent boys, presenting with acute unilateral and progressive orbital pain and swelling; the positivity of COVID-19 was determined by PCR test before surgery (90). Another case of sinusitis was also reported in a 76-year-old man who developed an orbital abscess with globe perforation. During surgery, avascular nasal mucosa was notable, which suggested thrombo-embolic complication of COVID-19 as the source of this event (91). Other orbital involvements, such as myositis (92) and emphysema (93), have also been reported.

A few cases of orbital involvement by SARS-CoV-2 have been reported, presenting within a median of 12 days. The virus's travel through the lacrimal pathway to the lacrimal gland during the early phase, as well as the hematologic spread of inflammation, can cause dacryoadenitis, as reported in one patient (94). Bilateral retro-orbital pain has been reported in another case, mimicking Deng fever (95).

### Quarantine dry eye

Patients under quarantine are at risk for ocular surface changes and developing dry eye disease (DED). Hence, the COVID-19 pandemic determines the elevation in patients with dry eye worldwide, a new event has been suggested called “quarantine dry eye,” following schools, universities, colleges, industries and offices were temporarily closed in most countries (96). There have been some suggestions for improving productivity for work or school models at home during quarantine, including smart work and online school lessons supported by video display terminals (VDTs). Such smart work models have resulted in the creation of new lifestyles, digital work and digital schooling, and limit young people and adults to long-term near-visual work. However, long-term use of VDTs may disrupt the surface of the eye, resulting in conditions such as DED (97).

This eye disorder has spread to the general population, even to children due to the widespread application of digital devices like tablets, computers, and electronic tools. While smartphone use is strongly associated with children's DED in urban settings, outdoor activities can offer a protective role. In this regard, DED may develop due to poor blink quality (mostly incomplete) or decreased blink rate (for example, due to enhanced exposure to blue light with short wavelengths) (98). Dry eye is associated not only with dietary consequences, but also with sleep deprivation and mental moods like anger and depression during COVID-19 pandemic (99). Quality of life can be reduced following quarantine dry eye, resulting in severe reduction in school and work efficiency. All of these bottlenecks can be overcome quickly with timely treatment of these conditions (98).

### Ocular surface route for COVID-19 transmission

Despite the possibility of ocular transmission of COVID-19 infection, the underlying mechanism remains questionable. The possibility of spreading coronavirus through tears is underestimated, but the virus may survive for a long time or multiply in the conjunctiva, even without symptoms of conjunctivitis, which indicates that eye protection (for example, goggles alone or in combination with face protection) is recommended to avoid contamination of external aerosols and droplets (100). A virus, including SARS, has been reported to infect host cells through angiotensin converting enzyme-2 (ACE-2) receptor and trans membrane protease serine-2 (TMPRSS-2) (101). Based on some experimental and clinical findings, SARS-CoV-2 has been isolated from tears and thus can be transmitted *via* this pathway (102). Despite the absence of the virus in the conjunctival sac of infected people without conjunctivitis and the low risk of transmission of coronavirus via tears, there is a possibility of long-term survival and conjunctival proliferation of SARS-CoV-2 after the symptoms of conjunctivitis disappear (15). Hence, it is necessary to protect the eyes (goggles alone or in combination with face shields) to prevent the risk of infection (100).

## Discussion

The COVID-19 infection is currently the most important health crisis in the world; although the respiratory system is the main organ involved during the disease, it has to be considered that other organs can also be affected by the SARS-CoV-2. Therefore, it is necessary to pay greater attention to any symptoms the patients may have. We discussed the ocular manifestations in the present review, some of which were supposed to be very rare at the first phase of the pandemic, but during the next phases and until today, ophthalmic and neuro-ophthalmic involvement has been reported more frequently, some of which are life- or organ-threatening and require urgent attention for appropriate diagnosis and management. Therefore, it is necessary for all physicians to know about the ocular manifestations of COVID-19, some of which present after recovery, in order to refer the patient on time to an ophthalmologist for accurate diagnosis and appropriate management (103).

As explained in the present review, ophthalmic, orbital, and neuro-ophthalmic complications are the three main categories, which require a thorough ophthalmic assessment to prevent undesired sequelae during or after COVID-19 infection. One of the limitations in the literature regarding the ocular presentations addressed in the present review is related to the small number of cases, which limits the suggestion of a causal relationship between the ocular presentations and COVID-19. Some ocular morbidities (such as exposure keratopathy, chemosis, and microbial keratitis), associated with critically ill patients, may be independent of COVID-19 infection and be related to the long period of ICU admission, use of sedation, and mechanical respiratory support (104, 105). Furthermore, the adverse effects of COVID-19 treatment are another cause of ocular complications during and after COVID-19. Chloroquine and hydroxychloroquine, prescribed for the patients with COVID-19 in higher doses than the safe dose, cause retinal toxicity, photoreceptor destruction, and bull's eye maculopathy (106). With the suggestion of anti-viral agents for the treatment of COVID-19, chloroquine and hydroxychloroquine were eliminated from the prescribed drugs, and different anti-viral medications have been suggested. However, the adverse effect of medications on the eye was not eliminated, as some of these anti-viral agents are also associated with drug-induced uveitis, transient myopia, and bilateral acute angle-closure glaucoma (secondary to the dopaminergic effect of Oseltamivir) (107).

The majority of the studies regarding COVID-19 and the ocular surface did not report/investigate the use of eye drops in these patients. However, a large number of ophthalmic medications have antiviral action. Antiviral adverse effects of numerous eye ointments and drops for various applications suggest that such secondary antiviral effects may be of particular importance in some viral infections (such as those with no valid treatment guideline) based on the reuse approach (108). Many of the eye preparations used for various eye diseases involve substrates with broad-spectrum intrinsic antiviral performance already clinically prescribed for other viral infections (other than COVID-19) (108).

The emphasis is on the positive effects of artificial tears and iodine or sodium hypochlorite eye drops to decline viral load on the surface of the eye by eliminating the virus or using a direct virucidal activity. The findings highlight the fact that ocular preparations provide a huge pool of potent candidates for re-use of the drug as antiviral therapies. Topical and systemic antiviral agents (eye drops or ointments) have been prescribed sparingly, and there are no specific antivirals for SARS-CoV-2-related ocular surface involvement (109).

Whether the eye is infected by the direct entry of the virus to the eye (through the expression of ACE2 receptors in some areas of the eye) or is affected indirectly by the systemic effects of the SARS-CoV-2 (thrombotic and inflammatory dysregulation) is not clear yet. However, as both routes are plausible and the ophthalmologists get close to the patient's nose, mouth, and tears during an ocular examination, it is necessary to follow the contact and droplet precautions during the examination, especially eye protection, for all health workers, especially ophthalmologists. The reports of transmission during ocular surgeries, such as cataract surgery, proposed the risk of infection during surgery, which reduced the number of some surgeries, such as corneal transplant surgeries, and caused most surgeons to perform only emergency surgeries during the COVID-19 pandemic.

Early in the COVID-19 pandemic and before widespread vaccination, the lack of adequate health and legal protection for surgeons and patients result in an excessive reduction in the volume of surgical interventions during a pandemic era. No specific protective regulations have been granted in many countries around the world to protect them from possible legal responsibility (110). In numerous articles presented in this pandemic time, the lack of universally agreed recommendations on safety systems and legal protection for ophthalmologists and eye surgeons can be seen. Healthcare professionals must take care to wear personal protective equipment (e.g. face shield) when examining those patients with ocular inflammation who are seen in the clinic (110). However, the academy of ophthalmology recommended protection for the mouth, nose (e.g., an N95 mask), and eyes (e.g., goggles or shield) and slit-lamp breath shields when caring for patients at a pandemic era (111). Therefore, precaution during surgery and the use of alternative strategies, such as telemedicine, are suggested to reduce the risk of virus spread (23).

## Conclusion

Ocular manifestations are uncommon features of COVID-19; however, some may be the presenting or the only symptom of the COVID-19 infection. Therefore, all physicians, especially ophthalmologists, should have a thorough understanding of the various ophthalmic manifestations in COVID-19 infection, both for early detection and appropriate management and following the necessary precautions to reduce the risk of transmission. Also, appropriate regulations and standard operative protocols should be implemented in clinical practice for the safety of the health care team and the patients to break the chain of transmission (104). On the other hand, there are several limitations among the studies that question the direct relationship of the ocular presentations with COVID-19, such as negative PCR results taken from the eye and the possible effect of underlying diseases, ICU admission, and medications used for COVID-19 treatment on ocular presentations. Thus, more studies are required in this regard. The issues addressed in the present review can help clinicians make better-informed decisions during the COVID-19 pandemic.

## Author contributions

MA: conceptualization, methodology, and writing—original draft preparation. MD: data curation. MA and MD: validation and writing—review and editing. All authors have read and agreed to the published version of the manuscript.

## Conflict of interest

The authors declare that the research was conducted in the absence of any commercial or financial relationships that could be construed as a potential conflict of interest.

## Publisher's note

All claims expressed in this article are solely those of the authors and do not necessarily represent those of their affiliated organizations, or those of the publisher, the editors and the reviewers. Any product that may be evaluated in this article, or claim that may be made by its manufacturer, is not guaranteed or endorsed by the publisher.
